# The Genetics of Adverse Drug Outcomes in Type 2 Diabetes: A Systematic Review

**DOI:** 10.3389/fgene.2021.675053

**Published:** 2021-06-14

**Authors:** Assefa M. Baye, Teferi G. Fanta, Moneeza K. Siddiqui, Adem Y. Dawed

**Affiliations:** ^1^Department of Pharmacology and Clinical Pharmacy, College of Health Sciences, Addis Ababa University, Addis Ababa, Ethiopia; ^2^Division of Population Health and Genomics, Ninewells Hospital and School of Medicine, University of Dundee, Dundee, United Kingdom

**Keywords:** pharmacogenomics, type 2 diabetes, adverse drug outcomes, oral glucose-lowering drugs, gastrointestinal side effects, hypoglycemia, weight gain

## Abstract

**Background:** Adverse drug reactions (ADR) are a major clinical problem accounting for significant hospital admission rates, morbidity, mortality, and health care costs. One-third of people with diabetes experience at least one ADR. However, there is notable interindividual heterogeneity resulting in patient harm and unnecessary medical costs. Genomics is at the forefront of research to understand interindividual variability, and there are many genotype-drug response associations in diabetes with inconsistent findings. Here, we conducted a systematic review to comprehensively examine and synthesize the effect of genetic polymorphisms on the incidence of ADRs of oral glucose-lowering drugs in people with type 2 diabetes.

**Methods:** A literature search was made to identify articles that included specific results of research on genetic polymorphism and adverse effects associated with oral glucose-lowering drugs. The electronic search was carried out on 3rd October 2020, through Cochrane Library, PubMed, and Web of Science using keywords and MeSH terms.

**Result:** Eighteen articles consisting of 10, 383 subjects were included in this review. Carriers of reduced-function alleles of organic cation transporter 1 (OCT 1, encoded by *SLC22A1*) or reduced expression alleles of plasma membrane monoamine transporter (PMAT, encoded by *SLC29A4*) or serotonin transporter (SERT, encoded by *SLC6A4*) were associated with increased incidence of metformin-related gastrointestinal (GI) adverse effects. These effects were shown to exacerbate by concomitant treatment with gut transporter inhibiting drugs. The CYP2C9 alleles, ^*^2 (rs1799853C>T) and ^*^3 (rs1057910A>C) that are predictive of low enzyme activity were more common in subjects who experienced hypoglycemia after treatment with sulfonylureas. However, there was no significant association between sulfonylurea-related hypoglycemia and genetic variants in the ATP-binding cassette transporter sub-family C member 8 (*ABCC8*)*/*Potassium Inwardly Rectifying Channel Subfamily J Member 11 (*KCNJ11)*. Compared to the wild type, the low enzyme activity C allele at CYP2C8^*^3 (rs1057910A>C) was associated with less weight gain whereas the C allele at rs6123045 in the *NFATC2* gene was significantly associated with edema from rosiglitazone treatment.

**Conclusion:** In spite of limited studies investigating genetics and ADR in diabetes, some convincing results are emerging. Genetic variants in genes encoding drug transporters and metabolizing enzymes are implicated in metformin-related GI adverse effects, and sulfonylurea-induced hypoglycemia, respectively. Further studies to investigate newer antidiabetic drugs such as DPP-4i, GLP-1RA, and SGLT2i are warranted. In addition, pharmacogenetic studies that account for race and ethnic differences are required.

## Introduction

Diabetes mellitus refers to a group of metabolic disorders characterized by hyperglycemia resulting from insufficient production and/or ineffective response of cells to insulin. It is one of the major contributors to morbidity and mortality globally, and its prevalence continues to rise. By 2019, an estimated 463 million adults aged 20–79 years were living with diabetes which accounts 9.3% of the global population in this age group (International Diabetes Federation, 2019). This figure is expected to surge to 578 million (10.2%) by 2030 and to 700 million (10.9%) by 2045, most of this in the form of type 2 diabetes (T2D) (International Diabetes Federation, 2019). Poorly controlled diabetes progressively leads to chronic microvascular, macrovascular and neuropathic complications which manifest as renal failure, blindness, lower limb amputation, and accelerated vascular disease.

Several drugs are available for the management of T2D, referred to as glucose lowering agents. These include: biguanides (metformin), insulin secretagogues (sulfonylureas, meglitinides), thiazolidinediones, alpha glucosidase inhibitors (acarbose), incretin mimetics (GLP-1RAs, DPP-4is), amylin antagonists, sodium-glucose co-transporter-2 inhibitors (SGLT-2i), and insulin. These classes of drugs are either prescribed as monotherapy or given in combination.

The management of T2D is guided by national and international evidence-based guidelines (Buse et al., 2020), but there is noticeable inter-individual variability in treatment response as defined by glycemic reduction and adverse drug reactions. This variation is the net effect of several environmental and clinical factors including age, sex, adherence, concomitant therapy, drug interactions, and disease severity. In addition to these, a patient's genotype can affect interindividual differences in drug response.

Adverse drug reactions are major clinical and public health problems world-wide. In the UK, around 6.5% of hospital admissions are due to adverse drug reactions (Pirmohamed et al., 2004), and that almost 15% of patients experience an ADR during their admission (Davies et al., 2009, 2010). The projected annual cost of such admissions to the NHS was £466m (Davies et al., 2009). Glucose lowering agents are one of the drugs of great concern for ADRs (Ducoffe et al., 2016). They have a well-described set of ADRs that are detrimental to individuals' health and quality of life (Table 1). In the US, from 2010 to 2013, there were 600,991 ADRs associated with glucose-lowering agents with an average hospital marginal cost of $4, 312 resulting in an annual hospital cost of $2.59 billion (Spector et al., 2017). Optimizing drug therapy through the avoidance of ADRs will dramatically improve patient health while generating millions of dollars by saving unnecessary medical costs.

**Table 1:** List of adverse drug reactions related to glucose-lowering drugs in type 2 diabetes.

**Drug**	**Side effects**	**Comments**
Metformin	GI side effects (10%−25%): nausea, indigestion, abdominal cramp or bloating, or combination of these (Bailey and Turner, 1996; Goodman, 2017). Decrease in vitamin B12 concentration (5 to 10%) (Bell, 2010; de Jager et al., 2010; Aroda et al., 2016; Donnelly et al., 2020) Lactic acidosis (Misbin, 2004)	Symptoms are usually mild, transient, and reversible after dose reduction. When severe requires drug switch in about 5% of the population. Patient may develop anemia and/or peripheral neuropathy (Bell, 2010). Rare but serious
Thiazolidinediones	Increased risk of overweight, congestion, heart failure, fractures, bladder cancer (pioglitazone) and myocardial infarction (rosiglitazone) (Jearath et al., 2016).	An average of 2 to 4 kg weight gain in the first year of treatment (Yki-Järvinen, 2004; Winkelmayer et al., 2008). Higher risk of adverse cardiovascular events with rosiglitazone (Winkelmayer et al., 2008; Juurlink et al., 2009; Graham et al., 2010).
Sulfonylurea and meglitinides	Hypoglycemia including coma (Schopman et al., 2014). Weight gain of 1–3 kg (Meneilly, 2011; Guardado-Mendoza et al., 2013)	
GLP-1 analogs	Nausea and vomiting, injection site reactions	Could be mediated through neural activation of specific CNS neurons due to peripheral dosing of peptide (Madsbad et al., 2011).
DPP-4 inhibitors	Headache, nasopharyngitis, and urinary tract infections (sitagliptin) (Katzung et al., 2019).	Have no effects on body weight or risk of hypoglycemia (Salvo et al., 2016).
SGLT2 inhibitors	Genital and urinary tract infections. IV volume depression and hypotension can result from osmotic diuresis (Katzung et al., 2019).	

However, there is inter-individual variability in the type and severity of ADRs experienced by individuals taking glucose-lowering drugs. While clinical and environmental factors influence this, genomic factors are also important. Here, we aim to undertake a systematic review of pharmacogenomic studies of ADRs related to oral glucose-lowering drugs.

## Methods

This systematic review is reported according to the Preferred Reporting Items for the Systematic Reviews and Meta-analysis Protocols (PRISMA-P) 2015 Checklist (Shamseer et al., 2015).

### Type of Participants

Participants included in eligible studies must be diagnosed with T2D and treated with oral glucose-lowering drugs.

### Type of Exposure

We included studies in which participants genotype were investigated in relation to ADRs of oral glucose-lowering agents.

### Outcomes

The primary outcome was the incidence of any of the adverse effects of glucose-lowering drugs (Table 1). For metformin, GI adverse effects were considered - hypoglycemia and weight gain for sulfonylureas and weight gain and edema for thiazolidinediones.

### Eligibility Criteria

#### Inclusion Criteria

Studies assessing the effect of genetic variations on the incidence of adverse effects of oral glucose-lowering drugs in people with T2D, published up to 3rd October 2020 in English language without any geographical restriction were included in this review.

#### Exclusion Criteria

We did not consider news, qualitative studies, case reports, reviews, editorials, and comments; and all non-published studies and published in non-English languages. Studies in which relevant data on genetic polymorphisms and/or ADRs associated with oral glucose-lowering agents is lacking or impossible to extract were also excluded.

### Search Strategy for Identifying Relevant Studies

A literature search was made to identify articles that included specific results of research on genetic polymorphisms and adverse effects associated with oral glucose-lowering agents. The electronic search was carried out on 3rd October 2020, through Cochrane Library, PubMed, and Web of Science using keywords and MeSH terms with no restriction for time of publication. The search strategy conducted in MEDLINE via PubMed, Cochrane, and Web of Science is shown in Supplementary Material. We also manually searched reference lists from relevant studies and contact experts in the field in order to identify additional eligible studies.

### Selection of Studies for Inclusion in the Review

Two review authors (AMB and AYD) independently identified articles and then screened their titles and abstracts for eligibility. Thereafter, full texts of articles considered to be eligible were retrieved. Furthermore, the review authors independently assessed the eligibility for inclusion in the review based on the inclusion and exclusion criteria. Disagreements between the two authors was resolved by consensus. A PRISMA flow diagram (Shamseer et al., 2015) was employed to document the process of literature selection and the reasons for exclusion of articles were stated.

### Data Extraction and Management

Two review authors (AMB and AYD) independently extracted data from the articles reviewed. A data extraction form for this purpose was designed. Data was collected on the first author's name, year of publication, geographical location (country where the study was performed), sample size, population characteristics, relevant genetic polymorphism, primary outcome measurements (incidence of adverse events after treatment with oral glucose-lowering agents). Any disagreements between the two review authors were resolved through discussion and consensus.

Study characteristics and the effect estimates of genetic polymorphisms on the incidence of adverse effects of oral glucose-lowering agents was presented in full, in tabular form. We summarized findings based on the type of oral glucose-lowering agent.

## Result

### Study Selection

**Figure 1:**
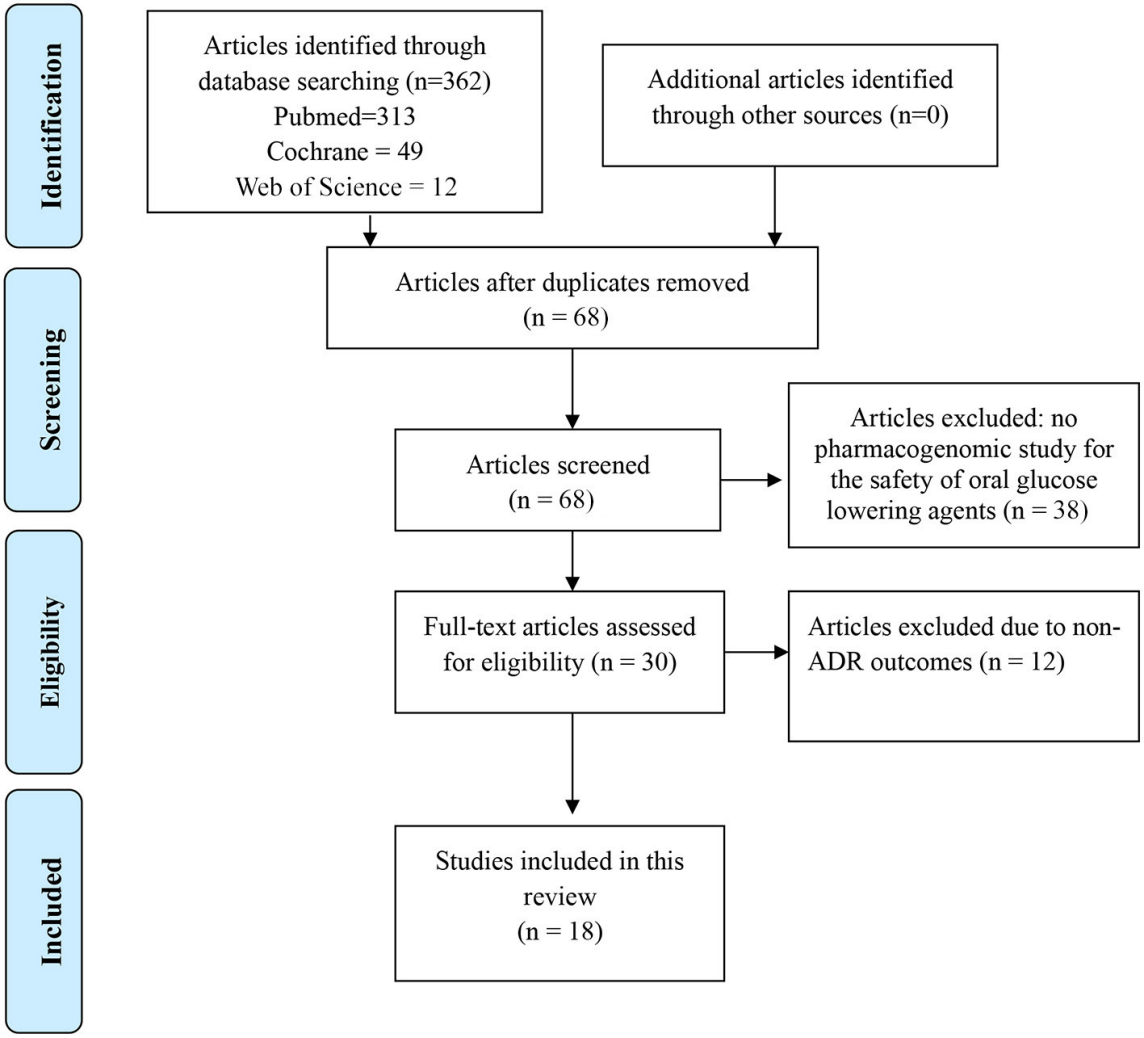
PRISMA flow diagram for search results October 30, 2020.

Of the 362 studies identified, 66 were included for review of the full text. Of these studies, 18 studies met the inclusion criteria (Figure 1).

### Characteristics of Included Studies

Eighteen articles comprised of 10, 383 subjects were included in this review (Table 2). Among these, two studies were multinational (Bailey et al., 2010; Dawed et al., 2019). While 12 studies were conducted in Europe (Holstein et al., 2005, 2011, 2012; Ragia et al., 2009, 2012, 2014; Tarasova et al., 2012; Dujic et al., 2015, 2016a,b, 2018; Dawed et al., 2016), three were in Asia (Kahn et al., 2006; Sato et al., 2010; Gökalp et al., 2011) and one was in the US (Ruaño et al., 2009).

**Table 2:** Description of included studies.

**References**	**Country**	**Study drug**	**Study period**	**Parent study**	**Population**	**Gene**	**Comparators**	***N***
Dawed et al. (2019)	Multinational (Europe)	Metformin		IMI DIRECT	White Europeans aged between 18 and 90 years	*SLC29A4*	Metformin-intolerant	286
							Metformin-tolerant	1,128
Dujic et al. (2015)	Scotland, UK	Metformin	1 January 1994–1 June 2011.	GoDARTS	White Europeans	SLC22A1	metformin-intolerant	251
							Metformin- tolerant	1,915
Dujic et al. (2016b)	Scotland, UK	Metformin	1 January 1994–1 June 2011.	GoDARTS	White Europeans	*SLC6A4* *SLC22A1*	Metformin-tolerant	1,356
							Metformin-intolerant	164
Dujic et al. (2016a)	Bosnia and Herzegovina	Metformin			T2D diagnosis after the age of 35 years	*SLC22A1*	Metformin-tolerant	49
							Metformin-intolerant	43
Tarasova et al. (2012)	Latvia	Metformin	from 2003 to 2010	LGDB	Subjects with T2D older than 18 years	*SLC22A1, SLC22A2, SLC47A1*	Metformin-tolerant	193
							Metformin-intolerant	53
Gökalp et al. (2011)	Turkey	Glimepiride Gliclazide Glipizide	2003 and 2005	None	Subjects with T2D treated with SU for at least 3 months.	*CYP2C9* *CYP2C19* *CYP2C8*	Without hypoglycemia	93
							With hypoglycemia	15
Dujic et al. (2018)	Scotland	Glibenclamide Gliclazide Glimepiride Glipizide	1994–2010	GoDARTS	Subjects with T2D who were incident users of SU	*POR* *CYP2C9*	Without hypoglycemia	311
							With hypoglycemia	69
Holstein et al. (2011)	Germany	Glimepiride Glibenclamide Gliquidone.	1 January 2000 and 31 December 2009	None	Subjects with T2D treated with SU	*CYP2C9*	Without severe hypoglycemia	101
							With severe hypoglycemia	102
Holstein et al. (2005)	Germany	Glibenclamide Glimepiride	January 2000 and 31 December 2003	None	Subjects with T2D treated with SU	*CYP2C9*	Without severe hypoglycemia	337
							With severe hypoglycemia	20
Holstein et al. (2012)	Germany	Glimepiride Glibenclamide Gliquidone	January 2000–31 December 2010	None	Subjects with T2D admitted to the emergency department	*ABCC8*	Without hypoglycemia	100
							With hypoglycemia	111
Sato et al. (2010)	Japan	Glimepiride Glibenclamide	January 2005 and October 2009	None	Subjects with T2D treated with Sus	*ABCC8*	Without hypoglycemia	32
							With hypoglycemia	125
Ragia et al. (2012)	Greece	Glimepiride Gliclazide	February 2007–September 2008	None	Subjects with T2D treated with SU	*KCNJ11*	Without hypoglycemia	84
							With hypoglycemia	92
Ragia et al. (2014)	Greece	Glimepiride Gliclazide	February 2007–September 2008	None	Subjects with T2D treated with SU	*CYP2C9*	Without hypoglycemia	84
							With hypoglycemia	92
Ragia et al. (2009)	Greece	Glimepiride Gliclazide	February 2007–September 2008	None	Subjects with T2D treated with SU	*CYP2C9*	Without hypoglycemia	84
							With hypoglycemia	92
Ruaño et al. (2009)	USA	Rosiglitazone Pioglitazone	Between February and June 2007	None	Subjects with T2D treated with rosiglitazone or pioglitazone for ≥4 months	**For BMI:** *ADORA1, PKM2, ADIPOR2, UCP2, APOH, IRS1, LIPA, RARB, and CHRM3* **For Edema:** *NPY, GYS1, CCL2, OLR1, GHRH, ADRB1, ACACB, SCARB2, HRH3 and ACE*		87
Kang et al. (2006)	South Korea	Rosiglitazone		None	Subjects with T2D aged between 35 and 80 years	*PLIN*		160
Dawed et al. (2016)	Scotland, UK	Rosiglitazone Pioglitazone			Subjects with T2D treated with TZD	*CYP2C8*		833
Bailey et al. (2010)	Multinational (21 countries)	Rosiglitazone Placebo		DREAM	Subjects with T2D ≥30 years of age	*NFATC2*	Rosiglitazone	965
							Placebo	956

Five of these studies comprising of 5,438 subjects were conducted to investigate association between genetic polymorphisms and metformin-related GI adverse effects (Tarasova et al., 2012; Dujic et al., 2015, 2016a,b; Dawed et al., 2019). Association between genetic polymorphisms and the risks of sulfonylurea-induced hypoglycemia was evaluated in nine studies comprising of 1,944 subjects (Holstein et al., 2005, 2011, 2012; Ragia et al., 2009, 2012, 2014; Sato et al., 2010; Gökalp et al., 2011; Dujic et al., 2018). Among the sulfonylureas, glimepiride was the most investigated (nine studies) (Holstein et al., 2005, 2012; Ragia et al., 2009, 2012, 2014; Sato et al., 2010; Gökalp et al., 2011; Dujic et al., 2018) followed by gliclazide (Ragia et al., 2009, 2012, 2014; Gökalp et al., 2011; Dujic et al., 2018) (five studies) and glibenclamide (Holstein et al., 2005, 2011, 2012; Sato et al., 2010; Dujic et al., 2018) (five studies). Four studies consisting of 3, 001 subjects evaluated the genetics of thiazolidinediones-induced edema and weight gain (Kang et al., 2006; Ruaño et al., 2009; Bailey et al., 2010; Dawed et al., 2016).

#### Metformin

Metformin is the first-line therapy for T2D. Around 30% of metformin treated subjects experience gastrointestinal (GI) side effects manifested as nausea, indigestion, abdominal cramps, bloating, diarrhea, or combination of these (Table 1) (Garber et al., 1997; Hirst J. A. et al., 2012). Metformin is an organic cation, and carrier proteins mediate its oral absorption, hepatic uptake, and renal elimination. Several solute carrier transporters, expressed in the membranes of the enterocytes, could be involved in the absorption of metformin from the intestinal lumen, including organic cation transporter (OCT) 1, plasma membrane monoamine transporter (PMAT), carnitine/cation transporter 1, OCT3 (encoded by *SLC22A3*), and serotonin reuptake transporter (SERT) (Han et al., 2015). Genetic variants in genes encoding these transporters have been reported in five articles (Table 3) (Tarasova et al., 2012; Dujic et al., 2015, 2016a,b; Dawed et al., 2019).

**Table 3:** Association between metformin and selected SNPs for the incidence of GI adverse outcomes.

**References**	**Outcome measure**	**Drug (*****n*****)**			**Gene**	**SNP/genotype**	**Conclusion**
		**Drug**	**Tolerant**	**Intolerant**			
Dawed et al. (2019)	Incidence of GI adverse effects	Metformin	1,128	286	*SLC29A4*	rs3889348G>A	The G allele at rs3889348 (*SLC29A4*) was associated with higher odds of gastrointestinal intolerance (OR 1.34 [1.09–1.65], *P* = 0.005).
Dujic et al. (2015)	Incidence of GI adverse effects	Metformin	1,915	251	*SLC22A1*	R61C C88R G401S M420del G465R	Compared to carriers of one or no deficient allele, carriers of two reduced-function OCT1 alleles had higher odds of intolerance (OR 2.41 [1.48-3.93], *P* < 0.001).
Dujic et al. (2016b)	Incidence of GI adverse effects	Metformin	1,356	164	*SLC6A4*	L*L* L*S* S*S*	Each S* alleles was associated with higher odds of metformin intolerance (OR = 1.28 [1.01–1.63], *P* = 0.04). Multiplicative interaction between *SLC6A4* and *SLC22A1* (*P* = 0.003)
					*SLC22A1*	R61C C88R G401S M420del G465R	
Dujic et al. (2016a)	Incidence of GI adverse effects	Metformin	49	43	*SLC22A1*	R61C M420del	Each OCT1 reduced-function allele was associated with higher odds of GI side effects (OR = 2.31 [1.07–5.01], *P* = 0.034).
Tarasova et al. (2012)	Incidence of GI side effects	Metformin	193	53	*SLC22A1* *SLC22A2* *SLC47A1*	rs12208357A>G rs34059508T>C rs628031A>G rs72552763D>I rs36056065I>D rs316019T>G rs2289669A>G	Each A allele at rs628031 was associated with lower odds of intolerance (OR = 0.39 [0.19–0.82], *P* = 0.01) Each 8 bp insertion at rs36056065 was associated with lower odds of intolerance (OR = 0.41 [0.23–0.72], *P* = 0.01)

Association between genetic variants in *SLC22A1* (a gene encoding OCT1), and GI intolerance related to metformin therapy have been reported in three studies (Tarasova et al., 2012; Dujic et al., 2015, 2016a). A study conducted using the GoDARTS cohort (Hébert et al., 2018), in 251 metformin-intolerant and 1,915 metformin-tolerant individuals showed that the presence of two or more reduced-function alleles at R61C, C88R, G401S, M420del, or G465R increased the odds of GI side effects of metformin by more than 2-fold (Dujic et al., 2015). This effect was over 4-fold with concomitant use of OCT1-inhibiting drugs. The findings were replicated in another prospective observational cohort study from Bosnia and Herzegovina that included 92 newly diagnosed subjects in the first 6 months of metformin treatment (Dujic et al., 2016a). Likewise, Tarasova et al. showed significant associations between a SNP (rs628031) and an 8 bp insertion (rs36056065) in the *SLC22A1*, with GI side effects of metformin (Tarasova et al., 2012).

Dawed et al. reported association between rs3889348, a variant that alters intestinal expression of the *SLC9A4* (a gene encoding PMAT), with metformin related GI intolerance in 286 severe intolerant and 1,128 tolerant subjects. The G allele that reduces expression of *SLC29A4* was associated with 34% higher odds of intolerance. Concomitant administration of metformin transporter inhibiting drugs exacerbate GI intolerance by more than 3-folds (Dawed et al., 2019).

Considering involvement of serotonin reuptake transporter (SERT) in metformin intestinal absorption, Dujic et al. investigated association between the low-expressing S^*^ allele derived from a composite SERT (*SLC6A4*)-5-HTTLPR/rs25531 genotypes and metformin intolerance. In this study, each S^*^ allele was associated with 30% higher odds of intolerance. Interestingly, a multiplicative interaction between OCT1 and SERT genotypes was observed (Dujic et al., 2016b). Carriers of reduced function alleles in OCT1 at the background of the wild type SERT (L^*^L^*^) genotype had greater odds of intolerance (OR 9.25 [3.18–27.0]) compared to carriers of the S^*^ allele.

#### Sulfonylureas

Sulfonylureas were the first oral glucose-lowering therapy introduced into clinical practice and along with metformin, are the most prescribed drugs for the management of T2D (Inzucchi et al., 2015). SU are transported into the liver by OATP1B1 (encoded by *SLCO1B1*) and metabolized mainly by the polymorphic CYP2C9 enzyme and to a lesser extent by CYP2C19 enzyme (Becker et al., 2008). CYP2C9^*^2 (R144C, rs1799853) and CYP2C9^*^3 (I359L, rs1057910) are the two most common variants that have been associated with poor metabolism of SU (Semiz et al., 2010). Sulfonylureas induce glucose-independent insulin release from the pancreatic β-cells by binding to the ATP-sensitive potassium (KATP) channels, SUR1 and Kir6.2, that are encoded by the *ABCC8* and *KCNJ11* genes, respectively.

Hypoglycemia is the most common adverse effect of SU. In a systematic review consisting of 22 randomized controlled trials, 10.1 and 5.9% of SU treated subjects experienced hypoglycemia as defined by blood glucose levels of ≤ 3.1 or ≤ 2.8 mmol/L, respectively (Schopman et al., 2014). Severe hypoglycemia with SU therapy is less common, with reported incidence of 0.8%. SU treatment also results in weight gain of 1–3 kg (Schopman et al., 2014). Risk of hypoglycemia and weight gain may vary with age, gender, renal function, disease progression, drug exposure, and genetic constitution.

In this systematic review, we have included studies that investigated association between genetic variants in genes, *CYP2C9* and *ABCC8/KCNJ11*, that encode CYP2C9 and SUR1/Kir6.2 with risk of hypoglycemia (Holstein et al., 2005, 2011, 2012; Ragia et al., 2009, 2012, 2014; Gökalp et al., 2011) (Table 4). An association between reduced function CYP2C9^*^2 and CYP2C9^*^3 alleles with higher risk of SU related hypoglycemia was reported confirming earlier functional and pharmacokinetic data (Ragia et al., 2009; Gökalp et al., 2011). However, another study could not confirm the findings (Holstein et al., 2011). In the later study subjects within the control arm that carry slow metabolizing alleles were found to be treated with significantly lower doses than carriers of the wild type, whereas in the group with severe hypoglycemia, the dose was the same for all genotype groups. Another small study suggested a possible interaction between P450 oxidoreductase (*POR*) and *CYP2C9* genotypes (Ragia et al., 2014), where POR^*^28 allele could mask the effect of CYP2C9^*^2 allele on sulfonylurea-induced hypoglycemia. Indeed, a bigger study from the GoDARTS cohort confirmed this and therefore it is worth considering *CYP2C9* and *POR* genotypes jointly in studies involving the pharmacogenetics of SU (Dujic et al., 2018).

**Table 4:** Association between sulfonylureas and selected SNPs for the incidence of hypoglycemia.

**References**	**Outcome measure**	**Sulfonylurea drug (%)**			**Gene**	**Genotypes**	**Conclusion**
		**Drug**	**Controls**	**Cases**			
Gökalp et al. (2011)	Incidence of mild hypoglycemia	Glimepiride Gliclazide Glipizide	44 (47%) 41 (44%) 8 (9%)	6 (40%) 5 (33%) 4 (27%)	*CYP2C9*	CYP2C9*2 CYP2C9*3	In the gliclazide group a significant association between *CYP2C9* genotypes and hypoglycemic attacks were observed (*P* = 0.035).
Dujic et al. (2018)	Incidence of SU-induced hypoglycemia	Glibenclamide Gliclazide Glimepiride Glipizide	5 (1.6%) 254(81.7%) 3 (1.0%) 49 (15.8%)	2 (2.9%) 53 (76.8%) 4 (5.8%) 10 (14.5%)	*POR* *CYP2C9*	POR*28 CYP2C9*2 CYP2C9*3	The number of *CYP2C9* deficient alleles increased the odds of hypoglycemia nearly 3-fold (OR, 2.81; 95% CI, 1.30-6.09; *P* = 0.009) only at the POR*1/*1 genotype background Statistically significant interaction between *POR* and *CYP2C9* genotypes (*P* = 0.007).
Holstein et al. (2011)	Severe hypoglycemia	Glimepiride Glibenclamide Gliquidone	81 (80.2%) 18 (17.8%) 2 (2%)	76 (74.5%) 25 (24,5%) 1 (1.0%)	*CYP2C9*	CYP2C9*1 CYP2C9*2 CYP2C9 *3	There was no overrepresentation of the CYP2C9 *2/*2, *2/*3, and *3/*3 variants in the SH group (2%) compared with the control group (5%).
Holstein et al. (2005)	Severe hypoglycemia	Glimepiride Glibenclamide	337	20	*CYP2C9*	CYP2C9*1 CYP2C9*2 CYP2C9 *3	The *CYP2C9* genotypes *3/*3 and *2/*3 that are predictive of low enzyme activity were more common in the hypoglycemic group than in the comparison groups (10 vs. <2%, OR 5.2; 95% CI, 1.01, 27).
Holstein J. D. et al. (2012)	Severe hypoglycemia	Glimepiride Glibenclamide Gliquidone	80 18 2	82 28 1	*ABCC8*	Ser1369Ala	Ser1369Ala variant in *ABCC8* does not affect the response to sulfonylurea treatment and so, is not a major player in the etiology of severe hypoglycemia.
Sato et al. (2010)	Severe hypoglycemia	Glimepiride Glibenclamide	32	125	*ABCC8*	Ser1369Ala	No significant differences in the distribution of the Ser1369Ala genotype between patients with or without severe hypoglycemia (*p* = 0.26)
Ragia et al. (2012)	Mild hypoglycemia	Glimepiride Gliclazide	4 10	80 12	*KCNJ11*	E23K	*KCNJ1*1 E23K polymorphism did not affect hypoglycemia risk.
Ragia et al. (2014)	Mild hypoglycemia	Glimepiride Gliclazide	74 10	80 12	*CYP2C9* *POR*	CYP2C9*1 CYP2C9*2 CYP2C9*3 POR*1 POR*28	POR*28 allele was not associated with severe hypoglycemia. CYP2C9 *2 allele increased the risk of hypoglycemia by more than 3 times (OR: 3.218, *p* = 0.031). POR*28 allele is masking the association of CYP2C9*2 allele with severe hypoglycemia.
Ragia et al. (2009)	Mild hypoglycemia	Glimepiride Gliclazide	74 10	80 12	*CYP2C9*	CYP2C9*1 CYP2C9*2 CYP2C9*3	The presence of CYP2C9*3 allele puts subjects with T2D at higher risk of hypoglycemia when receiving the SU

Three other studies investigated association between two strongly linked non-synonymous polymorphisms, S1369A (rs757110) and E23K (rs5219), in the *ABCC8* and *KCNJ11* genes, respectively with hypoglycemia (Sato et al., 2010; Holstein et al., 2012; Ragia et al., 2012). None of these studies showed statistically significant association between SU treatment and risk of hypoglycemia suggesting these polymorphisms may not play a major role in the etiology of hypoglycemia.

#### Thiazolidinediones

Thiazolidinediones are insulin sensitizers that act by increasing the transactivation activity of Peroxisome Proliferators Activated Receptors (PPARs). The clinically used TZDs, rosiglitazone and pioglitazone, suffer from serious side effects. Concerns about the cardiovascular safety of rosiglitazone due to fluid retention led suspension in the European market and several restrictions in the US (Woodcock et al., 2010; Shukla and Kalra, 2011). Unlike rosiglitazone, pioglitazone did not show any risk of cardiovascular side effects. However, concerns were raised on the apparent risk of bladder cancer with pioglitazone and hence it is not recommended in people with active or prior history of bladder cancer (Shukla and Kalra, 2011). TZDs are associated with an average of 2–4 kg weight gain in the first year of management (Yki-Järvinen, 2004). In addition, these agents result in peripheral edema in 4–6% (Graham et al., 2010).

This systematic review identified four articles that assessed association between genetic variants in candidate genes and weight gain and/or oedema after treatment with TZDs (Kang et al., 2006; Ruaño et al., 2009; Bailey et al., 2010; Dawed et al., 2016) (Table 5). A *post-hoc* analysis from the DREAM (Diabetes REduction Assessment with ramipril and rosiglitazone Medication) trial that consist of 4,197 participants showed higher rate of roziglitazone-induced edema (OR = 1.89 [95% CI = 1.47–2.42], *P* = 0.017) in subjects homozygous for the C allele at rs6123045, a variant at the Nuclear Factor of Activated T-cells, Cytoplasmic, Calcineurin-Dependent 2 (*NFATC2*) locus (Bailey et al., 2010). We have previously showed association between the CYP2C8^*^3 variant with less weight gain compared to the wild type (*P* = 0.02) in the GoDARTS (Dawed et al., 2016). Another study by Ruaño et al. investigated 384 SNPs in 87 subjects treated with TZDs and reported significant association between an intronic SNP, rs903361, in Adenosine A1 Receptor (*ADORA1*) and BMI after correcting for multiple testing (Ruaño et al., 2009). The A allele at Perilipin 1, PLIN 11482G>A (rs894160), was also associated with less weight gain in Korean subjects treated with rosiglitazone compared to the G allele (Kang et al., 2006). Given these findings replicate in well-powered independent studies, we could potentially identify individuals who can benefit from the considerable therapeutic advantages of TZDs and who are least at risk for the side effects.

**Table 5:** Association between thiazolidinediones and selected SNPs for the incidence of weight gain and edema.

**References**	**Outcome measure**	**Drug**	**Sample size**	**Gene**	**SNP/genotype**	**Conclusion**
Dawed et al. (2016)	Weight gain	Rosiglitazone Pioglitazone	519 273	*CYP2C8* *SLCO1B1*	CYP2C8*3 (rs10509681) 521T>C (rs4149056)	The CYP2C8*3 variant was associated with less weight gain (*P* = 0.02).
Bailey et al. (2010)	Edema	Rosiglitazone	965	GWAS	GWAS	rs6123045 an intronic SNP in the *NFATC2* was significantly associated with edema (OR 1.89 [95% CI 1.47–2.42]; *P* = 5.32 x 10^−7^)
Ruaño et al. (2009)	BMI	Rosiglitazone Pioglitazone	87	*ADORA1* *PKM2* *ADIPOR2* *UCP2* *APOH*	rs903361 rs2856929 rs7975375 rs660339 rs8178847	*ADORA1*-rs903361 was significantly associated with weight gain *P* < 0.0003)
Kang et al. (2006)	Weight gain	Rosiglitazone	160	*PLIN*	6209T>C 11482G>A 13041A>G 14995A>T	The A allele at 11482G>A was associated with less weight gain (GG, 1.33 ± 1.59 kg; GA, 0.85 ± 1.89 kg; and AA, 0.03 ± 1.46 kg; *P* = 0.010)

## Discussion

This systematic review considers the adverse effects of glucose lowering drugs and their relationship to genetic variability. It presents the up-to-date knowledge of genetic variants that could influence ADRs related to drugs for the management of type 2 diabetes. Comprehensive understanding of genetic variants associated with ADRs have clinical utility in risk stratification of patients and precision therapeutics. Although numerous associations of genetic variants with ADR have been discovered, replication has proven difficult. This could mainly be due to a smaller sample which may not be sufficiently powered to detect the desired effect, the lack of consistent phenotypic definitions used, presence of possible drug-drug interactions, and related comorbidities.

This systematic review suggests that at present the clinical translation of genetic variants associated with ADR in diabetes therapy are limited.

Inter-ethnic differences in the susceptibility to ADRs and response to drugs are under-investigated. However, inter-ethnic differences have long been recognized as a crucial aspect of the genetics of variation in drug response. This could be due to differing background frequencies of risk alleles. For example, the frequency of 420del allele, that causes reduced OCT1 function and associated with higher odds of GI side effects of metformin, is much higher amongst European (~17%) than East Asian (~0.5%) or African (~6%) populations (Karczewski et al., 2020). The same is true for the loss of function variant, CYP2C9^*^3 (European ~6.7%, African ~1.3%, South Asian 1.2%) (Karczewski et al., 2020).

It is also likely that adverse drug reactions are polygenic. In polygenic effects, risk is conferred by combinations between several variants each of which could have small individual effects that are summarized as polygenic risk scores. We have previously shown a better prediction of metformin-induced GI intolerance by combining SNPs in the OCT1 (*SLC22A1*) and PMAT (*SLC29A4*) (Dawed et al., 2019), pioglitazone-related weight gain and SNPs in the *SLCO1B1* and *CYP2C9* (Dawed et al., 2016). Comprehensive studies encompassing data from hypothesis free genome-wide associations are required to identify susceptibility loci. In addition, next generation sequencing that allows the analysis of rare variants that have been postulated to have larger effects are likely to reveal functionally relevant genomic variations for ADRs.

Clinical, anthropometric, and environmental factors such as age, sex, weight, concomitant use of other drugs was also shown to contribute to ADRs in diabetes. Older people, women, and concomitant use of gut metformin transporter inhibiting drugs were previously shown to increase the likelihood of GI side effects of metformin (Dujic et al., 2015; Dawed et al., 2019). In addition, longer diabetes duration, impaired renal function, lower body mass index, lower triglyceride levels and old age were identified as major risk factors for hypoglycemia in people with type 2 diabetes (Schloot et al., 2016).

Even though this review is comprehensive, it is subjected to limitations. First, the studies included were heterogeneous in design with regards to treatment, adverse effect outcomes definitions, and population (ethnicity). The timing to measure primary endpoint (adverse effects of oral glucose-lowering agents) is also not uniform.

Poor adherence to treatment is a well-known phenomenon in patients with diabetes and is associated with inadequate glycaemic control leading to rapid disease progression and complications (Polonsky and Henry, 2016). Moderate and severe ADRs such as hypoglycemia and GI intolerance are previously shown to be key contributors of poor adherence in diabetes.

In conclusion, there are few pharmacogenomic studies of ADRs in type 2 diabetes that have been undertaken. Most of the studies have not been externally replicated, except OCT1 and metformin induced GI intolerance, CYP2C9 and SU-induced hypoglycemia. In the future, well-powered pharmacogenomic studies in T2D should collect standardized ADR data in multi-ethnic populations.

## Data Availability Statement

The original contributions presented in the study are included in the article/Supplementary Material, further inquiries can be directed to the corresponding author.

## Author Contributions

AMB and AYD conceived and designed this research, executed the analysis procedure, and analyzed the results. AMB, MKS, and AYD contributed to the writing of the manuscript. All authors reviewed the manuscript.

## Conflict of Interest

The authors declare that the research was conducted in the absence of any commercial or financial relationships that could be construed as a potential conflict of interest.
