# Supplementary material for: The Genetics of Adverse Drug Outcomes in Type 2 Diabetes: A Systematic Review
Source: Front Genet. 2021 Jun 14;12:675053. doi: 10.3389/fgene.2021.675053 (PMC8236944; doi:10.3389/fgene.2021.675053)
Supplement: Supplementary file 1 [file Table_1.DOCX]

**Mesh terms**

“(Diabetes [MeSH Terms] OR type 2 diabetes) AND (genetic [MeSH Terms]) OR genetic markers OR genetic polymorphism OR Single nucleotide polymorphism OR polymorphism OR variant) OR gene OR allele) AND (hypoglycemic agents OR hypoglycaemic OR anti‐hyperglycemic OR anti‐hyperglycaemic OR antihyperglycemic OR antihyperglycaemic OR antidiabet* OR thiazolidinedione* OR pioglitazone OR rosiglitazone OR insulin secretagogue* OR sulfonylurea* OR sulphonylurea* OR glipizide OR glyburide OR glimepiride OR glibenclamide OR meglitinide* OR repaglinide OR nateglinide OR alpha‐glucosidase inhibitor* OR acarbose OR miglitol OR sodium glucose cotransporter OR empagliflozin OR canagliflozin OR dapagliflozin OR Dipeptidyl‐Peptidase IV Inhibitor* OR DPP4 inhibitor* OR saxagliptin OR sitagliptin OR alogliptin OR linagliptin OR vildagliptin OR Glucagon like peptide 1 analogs OR GLP-1 analogs OR exenatide OR liraglutide OR albiglutide OR Semaglutide OR lixisenaitide) AND (adverse effects [MeSH Terms] OR side effect* OR adverse drug reaction* OR adverse drug event* OR toxicity OR toxicities)
